# Treatment of Pulpless Teeth with Hydrogen Dioxide, Ethereal Solution

**Published:** 1898-04

**Authors:** George S. Allan

**Affiliations:** New York


### International Dental Journal.

Vol. XIX. April, 1898. No. 4.

### Original Communications.[1]

### TREATMENT OF PULPLESS TEETH WITH HYDROGEN DIOXIDE, ETHEREAL SOLUTION.[2]

BY GEORGE S. ALLAN, D.D.S., NEW YORK.

Notwithstanding the great amount of study and care taken by biologists and chemists, the production of an ideal germicide for stomatologists seems yet afar off. In medicine the chief uses for which a germicide or antiseptic is employed is either to keep pathogenic germs outside the system or from open cuts or wounds, or to provide a serum that will render the system immune to their hurtful action. So far as the teeth are concerned, for reasons not necessary to mention, the main want seems to be to find a germicide that will either utterly destroy the germs of putrefaction and fermentation, together with the decomposing contents of the pulp-chamber and tubules of so-called "dead teeth," or form with them some indestructible and inert compound, one that will not be a menace either to the safety or appearance of the teeth afterwards.

None of the organic germicides, such as creosote, carbolic acid, or any of the essential oils, will meet the conditions. Most of them are simply antiseptics, temporarily only inhibiting the growth of

[1] The editor and publishers are not responsible for the views of authors of papers published in this department, nor for any claim to novelty, or otherwise, that may be made by them. No papers will be received for this department that have appeared in any other journal published in the country.

[2] Read before The New York Institute of Stomatology, February 1, 1898.

germs. Very soon they become an additional source of danger, for in time they are acted upon by the germs and destroyed, and so add fuel, as it were, to the flames. A pledget of cotton, soaked with any one of the above-named agents and placed in the open cavity of a tooth, in a few hours becomes offensive and exhibits all the characteristics of decomposition and destruction. Of all the agents employed that will act upon dead tissues and form an indestructible compound, formaldehyde, I believe, is the most effective. I am uncertain, though, as to the duration of the compound that is formed,—that is, whether it is absolutely indestructible by the agencies in the mouth for any great length of time.

Formaldehyde is a gas that is held in solution in water. A forty-per-cent. solution is generally employed, but it is not permanent, for even if kept corked it is constantly losing its efficiency. In a preparation that I have had for a year or so in my office, one can to-day hardly detect the odor by placing it to the nostrils. And the odor is usually very strong and pungent. It is, however, a most valuable agent, is a real germicide, and is rapidly growing in favor.

The action of hydrogen dioxide is that of a destroying agent. It unites with the organic products of a dead pulp and absolutely destroys them as well as the germs themselves. The dead material is carried away with the gases generated. Hydrogen dioxide is simply oxygenated water. The symbol of water is $H_2O$. Hydrogen dioxide is $H_2O_2$. In other words, there is an extra atom of oxygen united with the two atoms of hydrogen. Pure hydrogen dioxide is a heavy liquid which is a chemical curiosity and most dangerous to handle. We know it only in its solutions,—either the aqueous or the ethereal solution. The two solutions have somewhat different properties, although acting on the same lines. The $H_2O_2$, in breaking up, becomes $H_2O$ plus the atom of oxygen liberated, and it is this atom of freshly liberated oxygen that does the work sought for. The ethereal solutions, five and twenty-five per cent., we are acquainted with are fairly permanent ones. The figures are accurate, and really represent the strength of the solutions. They are caustics, but their caustic properties are not injurious, and soon pass off. The treatment of a tooth containing a dead pulp with $H_2O_2$ is a very simple matter. The object desired is to bring it to an aseptic condition, and it is to this point more than all others that I wish to draw attention. The virtues of $H_2O_2$ as a bleaching agent have been heralded for several years, and in the hands of almost all good practitioners it has become an agent they cannot do without, its action being so quick, so certain, and so satisfactory to both operator and patient. But no attention, so far as I am aware, has been drawn to the fact that these teeth ought to be treated with $H_2O_2$, or with some other germicide, not alone to bring about the natural color, but also to restore the teeth to a healthy and, so far as possible, aseptic condition. I have been in the habit, since the first preparations were brought to our notice, of treating pulpless teeth, even molars and roots preparatory to capping, where discolorations would not be noticed, with $H_2O_2$ for the purpose of bringing them to a healthy condition, and the practice is proving very valuable. The treatment is, of course, the same that one would employ in bleaching: the apices of the roots being closed and the rubber dam applied when possible, the $H_2O_2$ is freely used.

As to bleaching I would say a word. Many use a twenty-five per-cent. ethereal solution and some a five-per-cent. for this purpose. Others still claim they obtain a more prompt and effective action by means of the electric (cataphoric) current. So far as I can see, there is nothing gained by using the cataphoric current for bleaching purposes. There is no advantage in using the twenty-five-per-cent. solution of hydrogen dioxide, and I always employ the five-per-cent. solution. If a five-per-cent. solution is used the evaporation soon reduces it to a twenty-five per-cent. solution, and by repeatedly swabbing out the cavity the desired change in color can be quickly obtained. I do not know that I have ever failed in my effort to bring a discolored tooth back to its normal color. It is well to be cautious and stop a little short of the full measure of success. Then seal a small quantity of the five-per-cent. solution in the tooth for a day, and usually no further treatment will be required. Bleaching can be, and often is, overdone. Great care should be exercised in guarding the adjoining teeth, if living, from the action of the agent. Even if the tooth be sound it may be affected, but if there happen to be an open cavity, there will surely be trouble and possibly intense pain, which may last for several days. A gold filling is only a partial preventive at best. To guard against these unpleasant sequences one must either isolate the tooth he is operating upon or else protect the adjoining teeth with a coating of wax or rubber in some way, so that the $H_2O_2$ will not come in contact with them.

Whenever it becomes necessary to remove the pulp from a tooth, the contents of the tubules also should be removed, for the latter are quite as likely to prove a cause of future trouble as is the pulp itself. In fact, it may safely be asserted that far too little attention has in the past been given to this point. It is only partly true that the dentinal fibres are hermetically sealed in the tubules after the pulp-chamber has been filled. The contents of pulp-chamber and tubules once removed, too great care cannot be taken to prevent the ingress of germs or germ foods in the future. $H_2O_2$ is, so far as I know, the only agent that will completely make way with everything objectionable.

In the treatment of deciduous teeth, when from the absorption of the roots root-filling is impracticable, an easy and reasonably sure method of practice is, after the removal of the pulp and contents of tubules, to first sterilize the pulp-chamber and tubules by using the three-per-cent. aqueous solution of hydrogen dioxide. The open ends of the tubules should then be closed with some preparation like cavatine, and the cavity and pulp-chamber only filled with gutta-percha or other soft filling-material. A temporary tooth carefully treated and filled in this manner seldom gives trouble, and the absorption of the roots is not necessarily interfered with.

"The most satisfactory method of obtaining absolute $H_2O_2$ for dental purposes is by immersing a pledget of absorbent cotton in pyrozone, twenty-five-per-cent. solution, and allowing the ether to evaporate. To prepare a fifty-per-cent. solution, it will be found most satisfactory to employ glycerin as the solvent. One tube of pyrozone, twenty-five-per-cent. solution, should be poured on fifteen minims of glycerin and allowed to stand in an open dish until the ether has evaporated. The glycerin will then contain fifty per cent. of $H_2O_2$. The uses of a solution of this strength will readily suggest themselves to the practitioner. Aqueous solutions of twenty-five-per-cent. strength can be readily prepared by shaking the contents of a tube of twenty-five-per-cent. solution with half a drachm of distilled water and separating the mixture through a funnel, or, better, by means of a pipette drawing off the ether floating on the top. The lower layer will contain the $H_2O_2$ in aqueous solution. Pyrozone, five-per-cent. solution ethereal, can be prepared by adding the contents of one tube of pyrozone, twenty-five-per-cent. solution ethereal, to half a fluidounce of pure sulphuric ether." It is interesting to note the behavior of the different aqueous solutions towards nitrate of silver. If there should be any hydrochloric acid present, its presence will be indicated by a more or less flaky precipitate. A faint opalescence however, does not indicate any acid reaction.

The twenty-five-per-cent. ethereal solution comes in small tubes hermetically sealed. They can easily and without any danger whatever be opened. It is wise to cool the tube before making the attempt. Then hold the tube wrapped in a wet towel, and gently cut the end with a file and break it off. Make the cut some distance back from the tip, and point the tube away from the face.

The most convenient vessels that I have found in which to make the aqueous solutions are the one-drachm (120-minim) glasses to be had at all druggists. Those with parallel sides are to be preferred. This aqueous solution, however, is not permanent. It can be kept for perhaps a week, but it gradually deteriorates and becomes cloudy and unfit for use, but as it is quickly prepared, one need not deny himself any advantages it may offer.

The aqueous and glycerin solutions of $H_2O_2$ do not seem to give the same amount of pain as the ethereal solutions, and this because the ether evaporates much more rapidly than the glycerin, and there would not probably be the same rapid disengagement of gas. I have within the last few days taken the twenty-five-per-cent. aqueous solution and swabbed out cavities with it in living teeth when excavating, without saying anything to the patient, and no notice was taken of it. I make these remarks with some caution, because I do not know what further experiments in this line may lead to, so that I would advise any one who thinks of using them to be cautious, because the pain that this $H_2O_2$ gives is greater than that caused by any other germicidal solution that we have; if we could get rid of that one objection, I think it would take the place of every other germicide for dental purposes.

Wishing to demonstrate the action of the ethereal solutions of $H_2O_2$, whether it would penetrate from the tubules through the cementum and to the outside of the tooth, I took this double root of a molar and coated the outside with wax, and also the exposed broken end, so that the only entrance for the $H_2O_2$ would be through the pulp-chamber; I then put it in a twenty-five-per-cent. ethereal solution and left it for an hour. As will be seen, the tooth, which was very much discolored and quite dark, is now restored to its normal color outside as well as inside, showing that the action of the $H_2O_2$ has been through the tubules and cementum, and that if the treatment had continued a great length of time there would have been pain in the socket of the tooth from the penetrating property of the gases through the whole thickness of the tooth to the peridentium. In bleaching teeth in the mouth I have many times obtained the same results. I have seen a portion of the roots exposed, stained dark yellow or brown, and restored to their natural color, the exposed portion of the root as well as the crown, and wholly from the action of the $H_2O_2$ in the pulp-chamber. Enamel is likewise penetrated by it through the dentine, but more slowly.

